# Xpert MTB/RIF Ultra for Tuberculosis Testing in Children: A Mini-Review and Commentary

**DOI:** 10.3389/fped.2019.00034

**Published:** 2019-02-28

**Authors:** Rachel R. Atherton, Fiona V. Cresswell, Jayne Ellis, Sabrina B. Kitaka, David R. Boulware

**Affiliations:** ^1^Infectious Diseases Institute, College of Health Sciences, Makerere University, Kampala, Uganda; ^2^Clinical Research Department, London School of Hygiene and Tropical Medicine, London, United Kingdom; ^3^LSHTM-MRC-UVRI Uganda Research Unit, Entebbe, Uganda; ^4^Hospital for Tropical Diseases, University College London Hospitals NHS Foundation Trust, London, United Kingdom; ^5^Department of Paediatrics and Child Health, Mulago Hospital and Makerere University School of Medicine, Kampala, Uganda; ^6^Division of Infectious Diseases and International Medicine, Department of Medicine, University of Minnesota, Minneapolis, MN, United States

**Keywords:** accuracy, sensitivity, Xpert, Ultra, TB, tuberculosis, children, pediatric

## Abstract

Tuberculosis (TB) remains a significant, yet under-recognized cause of death in the pediatric population, with a WHO estimate of 1 million new cases of childhood TB in 2016 resulting in 250,000 deaths. Diagnosis is notoriously difficult; manifestations are protean due to the high proportion of cases of extra-pulmonary TB in children, and logistical problems exist in obtaining suitable specimens. These issues are compounded by the paucibacillary nature of disease with the result that an estimated 96% of pediatric TB-associated mortality occurs prior to commencing anti-tuberculous treatment. Further development of sensitive, rapid diagnostic tests and their incorporation into diagnostic algorithms is vital in this population, and central to the WHO End-TB strategy. Initial gains were made with the expansion of nucleic acid amplification technology, particularly the introduction of the GeneXpert fully-automated PCR Xpert MTB/Rif assay in 2010, and more recently, the Xpert MTB/Rif Ultra (Ultra) assay in 2017. Ultra provides increased analytical sensitivity when compared with the initial Xpert assay *in vitro*; a finding now also supported by six clinical studies to date, two of which included pediatric samples. Here, we review the published evidence for the performance of Ultra in TB diagnosis in children, as well as studies in adults with paucibacillary disease providing results relevant to the pediatric population. Following on from this, we speculate upon future directions for Ultra, with focus on its potential use with alternative diagnostic specimens, which may be of particular utility in children.

## Background

Tuberculosis (TB) is currently the leading cause of mortality worldwide from a single infectious agent, being responsible for an estimated 1.7 million deaths in 2016 (1). In childhood, more than 96% of TB-related deaths are estimated to occur in children not receiving anti-tuberculous treatment, highlighting significant challenges in diagnosis.

The often non-specific nature of TB presentation in children has led to the development of a multitude of “scoring systems” based on clinical assessment and basic investigations, with most aimed at the diagnosis of pulmonary TB (2). However, weaknesses exist within these diagnostic algorithms. Many rely on the use of tuberculin skin testing, despite well-documented limitations, particularly in endemic areas. In addition, chest radiography is often recommended, but is difficult to interpret in children, who often do not demonstrate “classic” radiological findings indicative of TB (3). Due to the poor specificity of diagnostic algorithms, estimates have shown that TB may be both over-diagnosed and over-treated in some settings (4); yet underdiagnosed in other settings. Mycobacteriological diagnostics used in adults remain the “gold standard” but demonstrate a lower sensitivity in children (5), both from the paucibacillary nature of TB in children and the problem obtaining adequate respiratory or non-respiratory specimens for bacteriological confirmation (as young children are frequently unable to voluntarily expectorate sputum) (6).

The development of rapid diagnostic TB tests is recognized as a vital part of the WHO End TB Strategy (7), so as to allow appropriate early initiation of TB treatment and thus reduce mortality. Nucleic acid amplification tests have been available since the 1990s, and offer increased speed of detection when compared to mycobacterial culture, and increased sensitivity when compared to sputum smear. However, early iterations were costly and required a certain level of technical expertise to operate, limiting their generalizability in less economically developed settings.

### GeneXpert® MTB/Rif

Introduction of the first-generation cartridge-based NAAT GeneXpert® MTB/Rif (Xpert) assay in 2010 heralded the start of a revolution in TB diagnostics in low-resource settings. This fully-automated system has the advantage that it may be performed on-demand by personnel with minimal training with a run time of approximately 2 hours. In 2011, the WHO issued a strong recommendation for the use of Xpert assay as the initial diagnostic test for sputum samples from patients suspected of having pulmonary TB, to be used in preference to conventional microscopy and culture (8), followed in 2013 by a recommendation for use with cerebrospinal fluid (CSF) specimens from patients suspected of having TB meningitis (9), as well as lymph nodes and other tissues.

Multiple studies have added to the early evidence for the diagnostic accuracy of Xpert in pulmonary TB and rifampicin resistance in children (10) and adults (11). A recent meta-analysis of 15 studies (10), including 3,640 children, demonstrated a sensitivity of Xpert for TB detection of 62% using expectorated or induced sputum, and a sensitivity of 66% using samples from gastric lavage. Although this represented a 36–44% higher sensitivity when compared to smear microscopy, sensitivity remains poor when compared to an adult population [for which a recent Cochrane meta-analysis reported a pooled sensitivity of 89% for sputum Xpert (12)]. Indeed, one of the major recognized limitations of Xpert is a reduced sensitivity amongst key groups, including pediatric (10) and HIV-positive populations (12), due to the paucibacillary nature of disease.

### Xpert Ultra

In March 2017, following a multi-center non-inferiority study at 10 sites in eight low- and middle-income countries (13), Cepheid (with WHO approval) launched the second-generation GeneXpert® MTB/Rif Ultra (Ultra) assay (14, 15), with the aim of further increasing sensitivity. Two significant changes were made along with other technical optimizations. First, each cartridge included a larger chamber for DNA amplification, thus accommodating a larger-volume of sample proceeding forward into the actual PCR reaction. Second, two additional molecular targets for *Mycobacterium tuberculosis* were introduced, resulting in a decrease in the limit of detection from ~131 bacilli per ml of sputum for Xpert to ~16 for Ultra (15). Modifications were made only within the cartridge, allowing Ultra cartridges to be used with the pre-existing Xpert platform. The purchase price per Ultra cartridge remains the same ($9.98) as the original Xpert for countries eligible for concessional pricing.

We aimed to perform a mini-review and narrative of the evidence to date for the performance of Ultra in TB diagnosis in children, as well as studies in adults with paucibacillary disease providing results relevant to the pediatric population, followed by a commentary of future directions for Ultra.

## Materials and Methods

An electronic search was conducted with the aim of identifying all papers evaluating the accuracy of Ultra on any clinical specimen from adults or children with suspected tuberculosis. We searched the PubMed and Cochrane Library electronic databases for original studies and review articles published up to July 30, 2018, using the search terms: Xpert AND Ultra AND (tubercul^*^ OR tb). We excluded articles with title and abstract in any language other than English (0), and those for which we were unable to locate the full text (0).

We reviewed 15 full texts (all PubMed) and included 6 for discussion (13, 15–19) based on relevance of content to the review question (Table 1). Of the included texts, two evaluated the accuracy of Ultra for the diagnosis of pulmonary TB in the pediatric population (16, 17). Four additional studies evaluated Ultra in adult populations; (13, 15, 18, 19) with all including an assessment of participants with paucibacillary disease, providing further data relevant to a pediatric population.

**Table 1:** Characteristics of studies included in the review.

**Author**	**Site**	**Year**	**Type**	***N*; age/y**	**HIV /%**	**Prevalence /%**	**Sample type**	**Reference standard**	**Subgroup**	**n**_****P****_**/N**_****P****_ **SN/% (95% CI)**		**n**_****N****_**/N**_****N****_ **SP/% (95% CI)**	
										**Ultra**	**Xpert**	**Ultra**	**Xpert**
**CHILDREN**													
Nicol et al. (16)	South Africa	2012–2017	R	**367**; <15	19	23 (83/367)	Frozen sputum	Sputum culture; Xpert; Ultra	Total	56/76 **74** (62–83)	48/76 **63** (51–74)	225/233 **97** (93–99)	255/233 **100** (98–100)
									*HIV-positive*	*12/18* ***67*** *(41–87)*	NR	52/53 **98** (90–100)	NR
Sabi et al. (17)	Tanzania	2011–2012	R	**215;** 0.5–16	52	13 (28/215)	Frozen sputum	Sputum culture	Total	18/28 **64** (44–81)	15/28 **54** (34–73)	107/107 **100** (97–100)	105/105 **100** (97–100)
									*Smear-negative*	8/18 **44** (22–69)	5/18 **28** (10–54)	–	–
									*HIV-positive*	8/9 **89** (52–100)	6/9 **67** (30–93)	–	–
**ADULTS WITH PAUCILLARY DISEASE**													
Chakravorty et al. (15)	Peru, Vietnam, South Africa, Georgia, India	NR	R/P	**277**; NR	NR	72 (200/277)	Frozen sputum	Sputum culture	Total	NR **88** (82–92)	NR **81** (75–86)	NR **99** (93–100)	NR **99** (93–100)
									*Smear-negative*	NR/109 ***79** (70–86)*	NR/109 ***66** (56–75)*	–	–
Dorman et al. (13)	South Africa, Uganda, Kenya, India, China, Georgia, Belarus, Brazil	2016	P	**1439**; 21–59	25	32 (462/1439)	Sputum	Sputum culture	Total	408/462 **88** (85–91)	383/462 **83** (79–86)	934/977 **96** (94–97)	960/977 **98** (97–99)
									*Smear-negative*	86/137 ***63** (54–71)*	63/137 ***46** (37–55)*	–	–
									*HIV-positive*	*103/115* ***90*** *83–95*	*88/115* ***77*** *68–84*	–	–
Bahr et al. (18)	Uganda	2015–2016	P	**129**; 29–43	100	17 (22/129)	CSF	CSF culture; Xpert; Ultra	Total	21/22 **95** (77–99)	10/22 **45** (24–68)	101/106 **95** (NR)	NR
Perez-Risco et al. (19)	Spain	1999–2017	R	**168**; 19–89	NR	64 (108/168)	Frozen smear-negative samples	Sample culture	Total	82/108 **76** (67–83)	–	60/60 **100** (NR)	–

*Studies listed according to year of publication. R, retrospective; P, prospective; NR, not reported; SN, sensitivity; SP, specificity; CI, confidence interval; n_P_, number positive by both reference standard and Ultra/Xpert; N_P_, total number positive by reference standard; n_N_, number negative by both reference standard and Ultra/Xpert; N_N_, total number negative by reference standard*.

## Evidence for Use of Ultra in Children

The WHO endorse the use of Xpert as the initial diagnostic test in all children suspected of having TB, both for expectorated sputum and samples obtained via gastric lavage (9).

Two accuracy studies of Ultra exist in a pediatric population. The first was conducted by Nicol et al. (16), who retrospectively analyzed banked induced sputum from 367 children under the age of 15 years. The median age was ~3 years (interquartile range, 1.25 to 6 years). Of 76 microbiologically-confirmed TB cases using a composite reference standard of positive sputum Xpert, Ultra, or culture, sensitivity of Xpert was 63% (48/76, 95%CI 51–74), and sensitivity of Ultra was 74% (56/76, 95%CI, 62–83), representing an incremental benefit of 11%. Sensitivity of culture was 83% compared to the composite reference standard.

Specificity of Ultra was 97% (225/233, 95%CI 93–99); however, this rose to 100% when culture unconfirmed TB was included. The authors therefore argue that the lower specificity may represent misclassification of cases; indeed, the lower rate of prior TB infection in children (8.5% of this population had previously been treated for TB) likely decreases the number of false positive results caused by residual *M.tb* DNA from prior treated infection. Specificity of Ultra was lower in those who had been previously treated for pulmonary TB (96%, 23/34, 95%CI 79–100) when compared to those who were treatment-naïve (97%, 249/256, 95%CI 94–99), although not significantly so.

More recently, Sabi et al. (17) published the results of a multicenter diagnostic accuracy study, which examined frozen samples from 215 children across two sites in Tanzania, with a median age of 5.4 years (interquartile range, 1.5 to 9.9 years), and a higher HIV prevalence of 52%. 28 (13%) had culture-confirmed tuberculosis; in these patients sensitivity of Ultra was 64% (18/28, 95%CI 44–81) and sensitivity of Xpert was 54% (15/28, 95%CI 34–73) representing an 11% sensitivity increase similar to that of Nicol et al. Of note, both the above studies were retrospective, using cryopreserved sputum samples. Of the four studies conducted in adults, two also used cryopreserved samples (15, 19) whereas two were prospective (13, 18).

### HIV Co-infection Sub-group Analysis

Xpert or Ultra are currently recommended as the first-line investigation in all HIV-positive individuals, both adults and children. Nicol et al. included a sub-group analysis of HIV-positive children (*N* = 71) and reported a non-significant difference in Ultra sensitivity in the HIV-positive (67%) and HIV-negative (68%) populations. Sabi et al. also included a sub-group analysis, albeit with a smaller cohort of nine HIV-positive children, and reported a non-significant improvement (36%) in Ultra sensitivity in this population, when compared to HIV-negative children. When compared to the 19% improvement in Xpert sensitivity, these results suggest a greater additional diagnostic benefit for Ultra in the HIV-positive population, a result mirrored in the adult population (13).

## Evidence for Use of Ultra in Adults With Paucibacillary Disease

Further support for the utility of Ultra in children may be gleaned from studies of other pauci-bacillary populations, including smear-negative samples (13, 15, 19), as well as extra-pulmonary specimens (18, 19) in adults.

### Smear-Negative Pulmonary TB

Chakravorty et al. (15) performed a limited subgroup analysis of Ultra on 109 smear-negative specimens, as part of a wider assessment including 277 frozen sputum samples from a range of geographical settings. Using a reference standard of sputum culture, they demonstrated a sensitivity of Ultra for TB detection of 79% (95%CI 70–86) compared with 66% (95%CI 56–75) for Xpert in the smear-negative population.

Dorman et al. (13) also conducted a subgroup analysis using 137 smear-negative specimens, as part of a multicenter study in which sputum from a large cohort of adults (25% HIV-positive) with symptoms of pulmonary tuberculosis were evaluated using smear, mycobacterial culture, Xpert, and Ultra. Sensitivity in the smear-negative specimens was 63% (86/137, 95%CI 54–71) for Ultra compared with 46% (63/137, 95%CI 37–55) for Xpert, representing an increase of 17%. In comparison, across the whole study population, sensitivity of Ultra was 5% superior to Xpert (88 and 83% respectively) with both performing better than the 70% sputum smear sensitivity.

Dorman et al. also performed the most in-depth evaluation of the specificity of Ultra to date. Across all patients, specificity was lower for Ultra (96%; 934/977, 95%CI 94–97 compared to 98%; 960/977, 95%CI 97–99 in Xpert), an observation which may be largely attributable to detection of residual DNA or non-viable (non-culturable) *M. tuberculosis* bacilli from previously treated antecedent TB infection. This is consistent with the observation that specificity increased with increasing time since completion of treatment. The group also reported semi-quantitative Ultra results (including high, medium, low, very low, and a new “trace” category not available with Xpert). 44% of participants with a positive Ultra result had a “trace” result. Experts currently suggest that in persons with prior TB, trace results require confirmation by an alternative method.

### Extra-Pulmonary TB

The use of Ultra is also supported for specimens from certain patients suspected of having extrapulmonary TB, including CSF, lymph node aspirates, and other tissue specimens (9). For patients suspected of having TB meningitis, Ultra is recommended as the initial diagnostic test (9, 18). Limited evidence exists for use of Ultra and Xpert on other pauci-bacillary specimens.

Bahr et al. evaluated 129 HIV-positive adults in Uganda with suspected meningitis and assessed the performance of Xpert and Ultra against a composite reference standard based on positive CSF culture, Xpert, or Ultra (18). Amongst 22 patients with a microbiological TB meningitis diagnosis, sensitivity of Ultra was 95% (21/22, 95%CI 77–99) compared with 45% (10/22, 95%CI 24–68) sensitivity for Xpert or for culture. Although Ultra has a similar limit of detection *in vitro* as culture, Ultra has the added advantage of being able to detect non-viable *M.tb* bacilli in patients who have been initiated on anti-TB treatment in the days prior to sample collection (15). In high TB burden settings there can at times be a delay for several days, or even weeks, in performing lumbar puncture and TB treatment may be started whilst waiting for LP. In such cases molecular tests may be positive whilst culture remains negative. Unlike in sputum samples, false positivity of Ultra from residual *M.tb* DNA from prior treated TB is unlikely to be an issue in CSF, a sterile bodily fluid with high turnover, and TB meningitis usually only ever strikes once in a lifetime.

Further evaluation of Ultra sensitivity in extra-pulmonary specimens by Perez-Risco et al. tested 108 banked culture-positive, smear-negative specimens (19). HIV status was not recorded. Perez-Risco calculated specificities for a wide range of samples, both those already recommended by the WHO for use in conjunction with Ultra, as well as those currently not supported. Amongst supported specimens, sensitivity for gastric aspirate was 75% (3/4), CSF 100% (3/3); lymph nodes 94% (16/17); and other tissues was 87% (13/15). Non-supported extra-pulmonary samples were also tested. The sensitivities as follows were for urine 100% (12/12), joint fluid 88% (7/8), stool 80% (4/5), pericardial fluid 67% (2/3), abscess aspirate 65% (11/17), pleural fluid 48% (10/21), peritoneal fluid 33% (1/3) (19). However, the lower sensitivity of the latter samples likely related to the fact that only small aliquots were stored and tested. In order to optimize performance of Xpert or Ultra, large volumes should be concentrated by centrifugation (20). In testing 40 culture-negative samples and 20 non-tuberculous mycobacteria samples, they demonstrated a specificity of 100% (19). Although the numbers tested were small, the above study shows promise for the utility of Ultra in pauci-bacillary specimens. Still no test is 100% sensitive, including Ultra, thus a negative result cannot definitively exclude TB.

## The Future of Ultra in Pediatric TB Diagnosis

Over the past decade, significant advances have been made in TB diagnostics, characterized largely by the expansion of automated nucleic acid amplification technology to replace traditional sputum microscopy. Currently, focus remains on improving the technical performance of these tests, exemplified by the introduction of Xpert MTB/RIF Ultra as the next-generation Xpert, as well as expanding availability and faster turnaround time for test results in rural areas. We have reviewed the existing evidence regarding the accuracy of Ultra, with particular focus on the use of Ultra in children. Limited research has also been performed into improving the applicability of Xpert and Ultra, with particular regard to the use of alternative diagnostic specimens. However, further research is still required, and we conclude this review with an outline of the potential future directions for the use of Ultra in pediatric TB diagnosis, with a selection of key studies to date.

Despite the high morbidity and mortality associated with extra-pulmonary TB, most available tests are validated only for use on sputum. The use of alternative diagnostic specimens is of particular interest in the pediatric population, for whom sputum samples are notoriously difficult to obtain, and for whom extra-pulmonary disease constitutes approximately one-third of cases (21). However, there exists a relative paucity of diagnostics research using more easily available samples, such as urine, blood or stool. In a survey of 91 pediatric TB experts based in Europe in December 2017, 12.1% (*n* = 11) were already routinely testing stool samples using PCR-based assays, and 40.6% (*n* = 37) were routinely testing blood in suspected military TB, despite lack of evidence (22).

### Urine

Urine represents a clinical specimen that is both easily collectable and available in large quantities, and is therefore theoretically attractive as an extra-pulmonary sample for Ultra testing Currently, in patients with suspected genitourinary/renal TB, serial early-morning urine cultures are still considered the gold-standard. However, studies have examined the potential for using urine as a specimen for Xpert or Ultra in the diagnosis of genitourinary TB. A meta-analysis of Xpert use on urine, conducted across eight studies which included 725 specimens from subjects of all ages, demonstrated 70% sensitivity and 94% specificity for genitourinary TB compared with a reference standard of urine mycobacterial culture (23). As above, Perez-Risco et al. found Ultra to be 100% sensitive for culture-positive urine in adults (19), although patient numbers were small (*n* = 12).

However, urine is likely to be of less use in the diagnosis of pulmonary TB, as tuberculous bacilli must have disseminated to the urinary tract to be present, which is usually more common in immunocompromized patients (24). Whether Ultra could be used on urine as a useful adjunctive diagnostic in disseminated HIV-associated tuberculosis warrants investigation.

### Blood

The use of blood as a diagnostic specimen in pulmonary TB was evaluated in 44 HIV-positive adults with culture-positive sputum, with sensitivities for both liquid blood culture and blood Xpert reported as 21% (25). However, despite the low sensitivity of blood Xpert for pulmonary TB, the authors note positivity to be highly predictive of early mortality, with potential utility for Xpert as a prognostic indicator, to aid in distinguishing patients who may benefit from more intensive treatment or immunomodulatory therapy.

### Stool

Stool represents a more promising specimen for suspected pulmonary TB, as *M. tuberculosis* may be present from swallowed sputum. However, in a study of 37 children with bacteriologically-confirmed TB, stool Xpert demonstrated a sensitivity of only 29.7% although Xpert was superior to the 13.5% stool smear or 16.2% stool culture sensitivity (26). As Ultra has an eight-fold lower limit of detection (~15 CFU/ml vs.~100–120 CFU/ml for Xpert), Ultra may be a promising assay for use on stool, which can be readily collected from any infant mitigating the need for gastric aspirate. A recent evaluation of Ultra showed a sensitivity of 80% amongst five stool culture-positive adults (19); this certainly warrants further investigation in the pediatric population.

Currently, Xpert is not recommended by the WHO using any of the above samples, of urine, blood, or stool due to a lack of adequate evidence (9). Further prospective evaluation of the diagnostic performance of Ultra on non-respiratory specimens is needed, particularly in the pediatric population.

Further work is also required in evaluating the performance of Ultra alongside other innovative diagnostic tests, such as the TB lipoarabinomannan (TB-LAM) lateral flow assay (Alere) and a near future second-generation TB-LAM, and as part of the diagnostic algorithms in a wide range of patient populations. Moreover, currently no evidence exists for the potential of novel diagnostics to reduce morbidity or mortality in childhood TB (27). Evaluation of the impact of Ultra in increasing diagnostic yield, reducing time to treatment initiation and improving outcomes, will be helpful programatically. Finally, previous studies which have performed cost-benefit analysis of Xpert (28), both in low-burden and high-burden countries (29–32); should be replicated for Ultra, across different geographical and economic settings.

## Conclusions

TB still poses significant diagnostic challenges in children, and the development of further diagnostic options is essential in reducing mortality and morbidity from this condition. Ultra represents the most recent advancement in molecular diagnostics and is recommended by the WHO for the diagnosis of pulmonary TB in children, in conjunction with sputum or gastric aspirate samples. Ultra boasts increased sensitivity compared to its predecessor Xpert, with particular gains demonstrated pauci-bacillary populations. The slight reduction in specificity seen in adult studies with previously treated TB is less likely to represent a problem in TB-naïve pediatric populations. A challenge in children remains the difficulty obtaining suitable diagnostic specimens. Studies have shown potential for use of Ultra in alternative samples, particularly stool. Further prospective evaluation is required.

## Author Contributions

DB devised the review, the main conceptual ideas, and proof outline. RA drafted the manuscript and FC, JE, SK, and DB revised the manuscript critically for important intellectual content. All authors approved of the version of the manuscript to be published.

### Conflict of Interest Statement

The authors declare that the research was conducted in the absence of any commercial or financial relationships that could be construed as a potential conflict of interest.
